# Supplementary figures and images for: Natural selection exerted by historical coronavirus epidemic(s): comparative genetic analysis in China Kadoorie Biobank and UK Biobank
Source: BMC Genomics. 2025 Oct 21;26:943. doi: 10.1186/s12864-025-11876-4 (PMC12542218; doi:10.1186/s12864-025-11876-4)

**
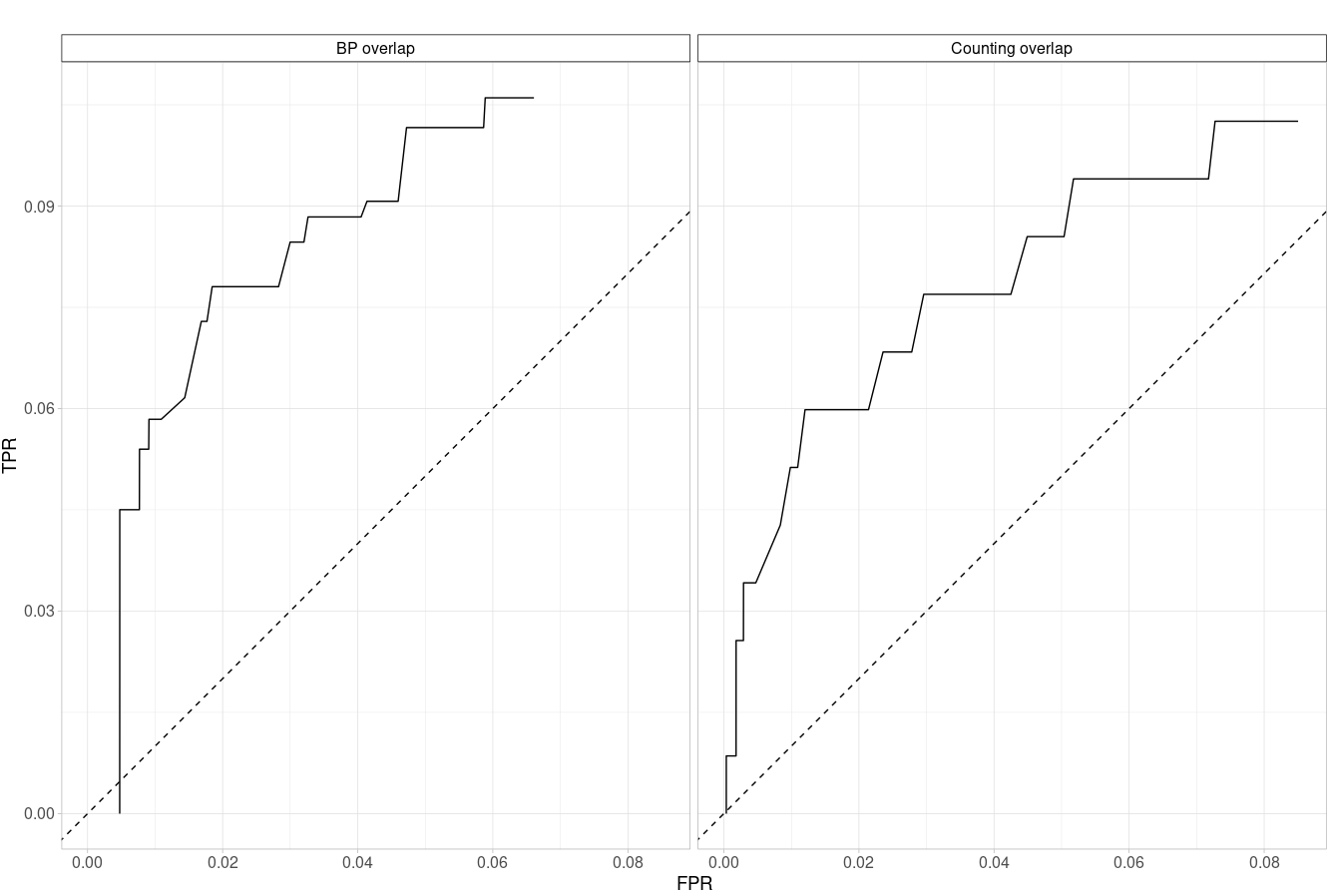
Supplementary Figure 1**.

Supplement: Supplementary file 3 — Supplementary Material 3. Supplementary Figure 1. Receiver Operating Characteristic (ROC) curves comparing the performance of Long Range-LD selection regions, using saltiLASSI selection regions as the truth set. The left panel (labelled "BP overlap") evaluates performance based on summing the total number of overlapping base pairs, while the right panel (labelled "Counting overlap") evaluates performance based on counting overlapping regions. The True Positive Rate (TPR) is plotted against the False Positive Rate (FPR) for different thresholds, with a dashed diagonal line representing the performance of a random classifier for comparison. [file 12864_2025_11876_MOESM3_ESM.docx]
